# Identification of carbon nanotube particles in liver tissue and its effects on apoptosis of birds exposed to air pollution

**DOI:** 10.14202/vetworld.2019.1372-1377

**Published:** 2019-09

**Authors:** Ahmed Mahdi Al-Badri, Ali Fayadh Bargooth, Jafar Ghazi Al-Jebori, Esraa Abdul Khaliq Zegyer

**Affiliations:** 1 Department of Biology, College of Science, Wasit University, Wasit, Iraq; 2 Department of Biology, College of Education for Pure Sciences, Wasit University, Wasit, Iraq; 3 Department of Anatomy and Histology, College of Veterinary Medicine, Al-Qasim Green University, Babylon, Iraq

**Keywords:** air pollution, apoptosis, carbon nanotube

## Abstract

**Aim:**

This study aimed to distinguish carbon nanotube (CNT) particles and their pathological effects on the liver of birds in areas with carbon emissions.

**Materials and Methods:**

Twenty-one domestic ducks were collected from pure farmers and exposed to different sources of air pollution. Histological stains were used to detect the accumulation of carbon particles. In addition, acridine orange/ethidium bromide staining was used to detect apoptosis, and scanning electron microscope (SEM) technique was used to determine the morphological design of carbon particles.

**Results:**

Light microscope results showed that the liver sections contain multiwalled CNTs (MWCNTs) which appear as black spots in the hepatic parenchyma. The histopathological changes of parenchyma include sinusoidal dilatation, infiltration, and congestion with frequently high number of macrophages. In general, early destruction of hepatic parenchyma was observed. Moreover, SEM results showed two morphological types of CNTs: The ball-shaped nanoparticles scattered as ultrafine carbon black and fiber form of carbon particles were recognized as MWCNTs in the hepatic tissue. Fluorescence microscopy results showed the early and progressive stages of apoptosis in the hepatic cells of birds in polluted areas, which can be related to the degree and exposure period to pollutants.

**Conclusion:**

The study indicates that liver morbidity of birds living in the farms affected by the pollution of brick factories is higher than the birds living in farms affected by the pollution of oil fields.

## Introduction

Atmospheric pollutants are the most widespread entities that have been exerting harmful consequences on the global ambient environment [1]. The range of ambient carbon dioxide levels was 300-700 ppm in some studies. At above this range, some occupants began to show one or more of the standard symptoms of carbon dioxide poisoning such as hyperventilation, rapid pulse rate, and difficulty in breathing, headache, sweating, and fatigue [2]. Birds are more susceptible to exposure to particle pollution than mammals due to higher rates of respiration and spending more time in the open air. The atmospheric particulate material is greatly problematic to birds due to their thinner capillary lungs, which subsequently makes them highly vulnerable [3]. The feeding activity of some birds which are well-known to feed on nectar, like the ruby-throated hummingbird (*Archilochus colubris*), could be influenced by earth and ozone level of atmospheric pollutants. In addition, these birds breathe greater volumes of air contrary to their low body weight [4]. Some types of birds like sparrows that live in highly polluted urban zones suffer from remarkably reduced antioxidant capacities and hemoglobin contents. On the other hand, the extent of this effect depends on the level of pollution present in bird habitats [5]. Long-term exposure to air pollutants can cause remarkable body weight loss, inflammation, and lung failure which also lead to lower red blood cell counts and blood vessels rupture [3]. In Beijing and Manila, it has been found that air pollution can result in black lungs and enlarged testicles in birds. In addition, the livers of Beijing birds were found to have more polycyclic aromatic hydrocarbons through deposits of fossil-like burnings, compared to birds living in a healthy air quality environment [5]. Carbon nanotubes (CNTs) are a very desirable nanomaterial in a wide variety of applications due to their excellent electrical and mechanical properties. However, its apprehensions have been elevated about safety due to their chemical constancy and structural resemblance to asbestos fibers [6]. CNT characterization and its impact on human tissues have been studied. The morphology of CNTs and their aggregate state appeared as single-walled CNTs, multiwalled CNTs (MWCNTs), and ultrafine carbon black (UFCB) which is employed by scanning electron microscopy (SEM) [7].

In a number of animal studies, the attention has been strengthened; respiratory tract exposure to CNTs has shown a very toxicological response described as granulomas and fibrosis inflammation with little or no-effect levels [8]. Cells make up the basic structure of all organisms and creatures. Both their proliferation and death must be forcefully regulated to maintain normal function of organs. Several cell death mechanisms are well-known in body tissues including apoptosis, necrosis, necroptosis, and autophagic-dependent cell death [9]. The term “apoptosis or programmed cell death” is currently used to refer to a mechanism used by biological organisms to assure their normal development and homeostasis of body organisms [10]. Furthermore, apoptosis has several pathogenic roles in different dreadful diseases including cardiomyopathy, acquired immunodeficiency syndrome, and cancer [11]. In recent years, methods of apoptosis detection have been studied abundantly in biological sciences. Research is massively being carried out to recognize the regulation of apoptosis in the ovary [12] and different extragonadal cell systems [13]. There are many studies in poultry focused on determining the relationship between apoptosis and damage of different tissues including myocytes in heart, lung tissues, hepatocytes in the liver, white and red pulp in spleen, and glomerulus in kidneys [14]. Some authors have summarized the mechanism of hepatocyte apoptosis including the classical intrinsic and extrinsic apoptotic pathways, the oxidative stress, and endoplasmic reticulum stress-induced apoptosis. They have emphasized the main reasons of apoptosis according to the characteristics of different liver diseases [15].

However, a limited number of studies have reported the identification of CNT particles in liver tissue. In addition, few studies have defined the relationship between its effects on apoptosis of birds exposed to air pollution. Hence, this study attempts to investigate the relative concentration and morphologies of agglomerated CNT particles in the liver of birds living in soot-polluted areas through using light microscope (LM) and SEM technique. Subsequently, it focuses on illustrating how CNTs can cause apoptosis using fluorescent technique.

## Materials and Methods

### Ethical approval

This research was conducted under the supervision of Wasit University Research Council and the approval of the College of Science/Department of Biology. All animal experiments were performed according to global animal ethics and under the supervision of several specialists at Wasit University.

### Animals and tissue preparation

Twenty-one domestic ducks were collected from different farms of Wasit Province, Iraq. These birds were divided into two groups according to the direct exposure to air pollution, including long-term air pollution area (areas: oil fields and brick factories) and the area with clean air. Each group contained seven birds and samples were clinically healthy and without any injuries. Body weight was measured (Table-1) and all the animals were sacrificed by cervical dislocation under the supervision of Animal House Veterinary Staff and according to the university policies. Then, the liver samples were extirpated completely from the abdominal region. Finally, all the samples were studied using the light and electron microscope techniques.

**Table 1:** The mean body weight (g) in three groups of birds.

Living areas of birds	Body weight (Mean±SE)
Clean	1592±110.43
Oil fields	1459±141.83
Brick factories	1652±66.07

SE=Standard error, n=7 and P≤0.05 between different areas

### LM technique

For histological study, first, several samples of the liver were fixed in 10% neutral buffered formalin for 72 h and washed with tap water for 2-3 h. Then, these samples were briefly transferred to several histological technique steps including dehydration, clearing, infiltration, embedding, and cutting. Serially, sections at 5-7 μm thickness were stained by freshly filtered hematoxylin and eosin (H and E) stains and periodic acid Schiff (PAS) staining method to reveal the general structure of the tissue in addition to detecting precipitate CNT particles in samples paraffin sections. Finally, the stained sections were mounted on a slide with a coverslip [16].

### Fluorescent microscopy technique

To detect and determine apoptosis, in this study, the apoptosis in liver tissue was quantified by fluorescence microscopy after treating by a mixture of acridine orange (AO) with the concentration of 10 µg/ml and ethidium bromide (EB) with the concentration of 10 µg/ml. Paraffin blocks were sectioned at 6 μm thickness, then slides deparaffinized in two changes of xylene for 5 min each. The sections were rehydrated in ethanol alcohol (99%, 90%, and 70%) and transferred to distilled water. Next, AO/EB stains were added to the slides for 10 min and washed with phosphate buffer saline to eliminate stains; then, they were dried and mounted with DPX media. Apoptotic cells were recognized by their shrinking, budding, and absent cytoplasm and fragmented nuclei and fluorescence enhancement by condensed chromatin. When the stain was associated with DNA, the AO changed the color of cells to green; however, the cells in the late stage of apoptosis turned orange-red.

### SEM technique

SEM technique was used for observing the accumulation of CNT particles in liver tissue. The samples were isolated from sacrificed birds and were fixed in 1% glutaraldehyde and 3% paraformaldehyde in a buffer of 0.1 M phosphate buffer (pH 7.3) for 24 h and, subsequently, were fixed with 1% osmium tetroxide in the same buffer for 2 h. After dehydrating in an ethanol series, drying by critical point method, and coating the sample with a layer of gold, the samples were examined by SEM technique.

## Results

### LM results

**Figure-1:**
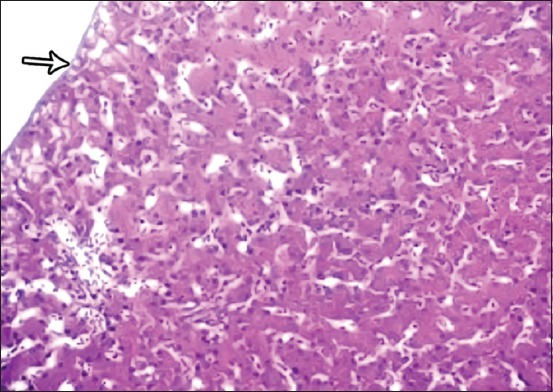
Photomicrograph of liver showing thin capsule (Glisson’s capsule) (arrow) surrounded by the hepatic structure. Hematoxylin and eosin, 40×.

**Figure-2:**
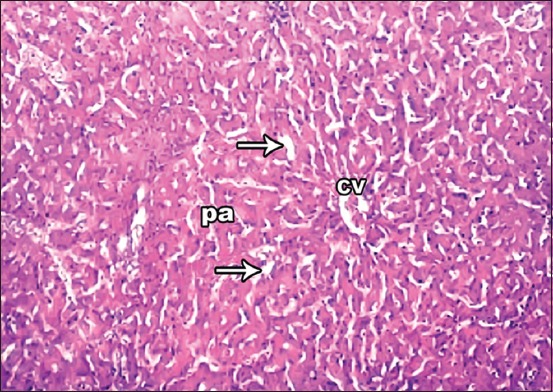
Photomicrograph of liver showing hepatic lobules central vein, portal area, sinusoid (arrow). Hematoxylin and eosin, 40×.

**Figure-3:**
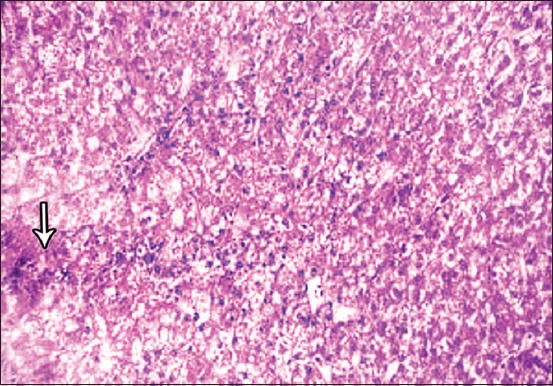
Photomicrograph of liver sections showing (black arrow) indicate multiwalled carbon nanotubes deposition in liver interstitium. Hematoxylin and eosin, 100×.

**Figure-4:**
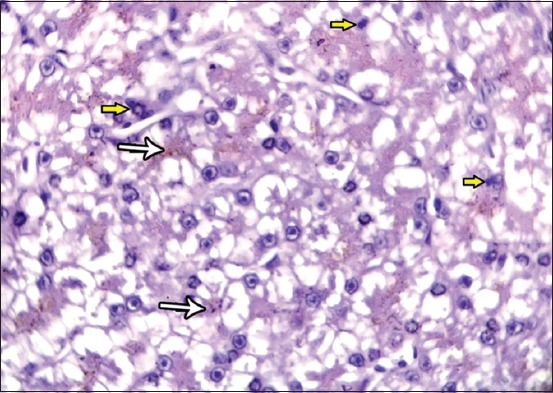
Photomicrograph of liver sections showing the liver sinusoids appears as congestion (black arrow) and pervaded with macromolecular of carbon nanotubes and Kupffer cells (yellow arrow). Periodic acid Schiff, 1000×.

**Figure-5:**
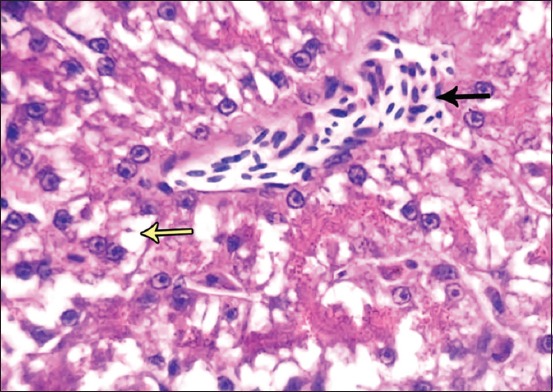
Photomicrograph of liver sections showing histopathological changes including sinusoidal infiltration (black arrow) and vacuolization (yellow arrow). Hematoxylin and eosin, 400×.

**Figure-6:**
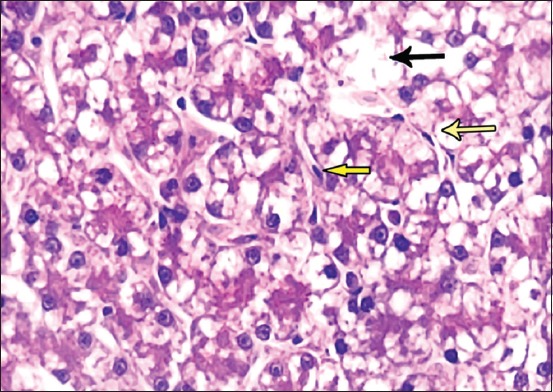
Photomicrograph of liver sections showing sinusoidal dilatation (black arrow) and flattened macrophage cells (yellow arrow). Hematoxylin and eosin, 400×.

The histological examination by H and E stains and PAS staining showed the general structure of liver in the healthy domestic ducks. The parenchyma of liver surrounded by a thin capsule (Glisson’s capsule) (Figure-1), which mainly composed of collagen fibers and smooth muscle fibers was present. The principal cells of the liver are hepatocytes, organized with the stromal elements of the liver lobule as an anatomical unit. The hepatocytes arranged as sheets, separated by the hepatic sinusoids (Figure-2). The histological examination of liver samples of birds living in polluted areas (both areas of oil fields and brick factories) showed that the sections contained MWCNTs, which appear as black spots in the parenchyma of liver (Figure-3). The liver sinusoids appear as congestion pervaded with micromolecule of CNTs; these sinusoids frequently contain a high number of the macrophages, Kupffer cells which are most prominent in vascular architecture (Figure-4). The results showed that the few histopathological changes of parenchyma include sinusoidal dilatation and infiltration (Figures-5 and 6). As to the toxic effect of the carbon components, early destruction of hepatic parenchyma occurs, which assimilates the damage to the extracellular matrix and vacuolization in hepatocyte (Figure-5); it also stimulates activation of hepatic stellate macrophage cells flattened to extend the perisinusoidal space (Figure-6).

### SEM results

**Figure-7:**
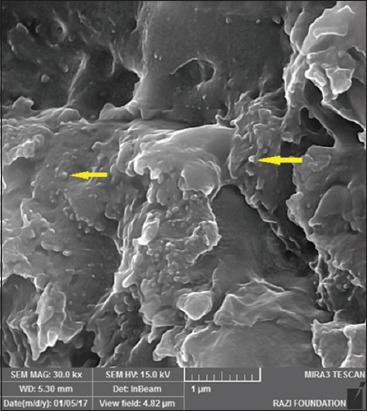
Electron microphotograph of liver showing ultrafine carbon black (yellow arrows). X 3500.

**Figure-8:**
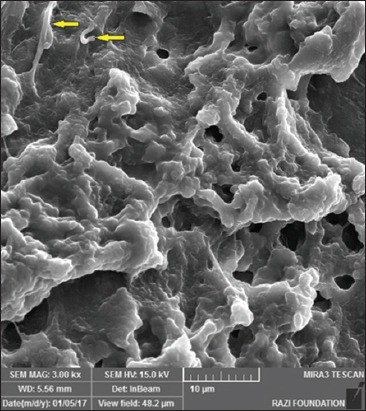
Electron microphotograph of liver showing multiwalled carbon nanotubes (yellow arrows). X 1000.

The SEM results of liver samples taken from the polluted areas showed two forms of CNTs: The widespread ball-shaped nanoparticles were scattered as UFCB (Figure-7) and the fiber form of carbon particles was recognized as MWCNTs in the hepatic tissue (Figure-8). All SEM results were in conformity with the findings of LM.

### Fluorescent microscopy results

**Figure-9:**
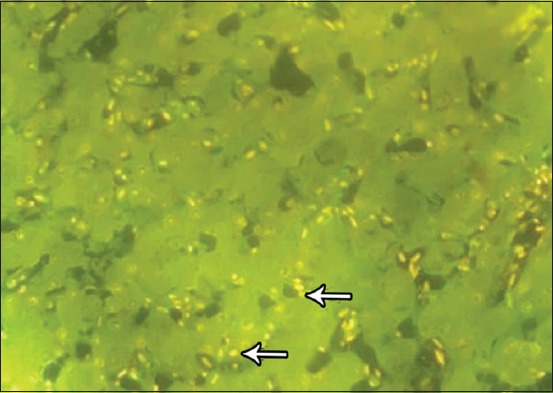
Fluorescence microscopy investigation of hepatic tissue showing the early apoptosis as yellowish color (yellow arrows). Acridine orange and ethidium bromide stain, 100×.

**Figure-10:**
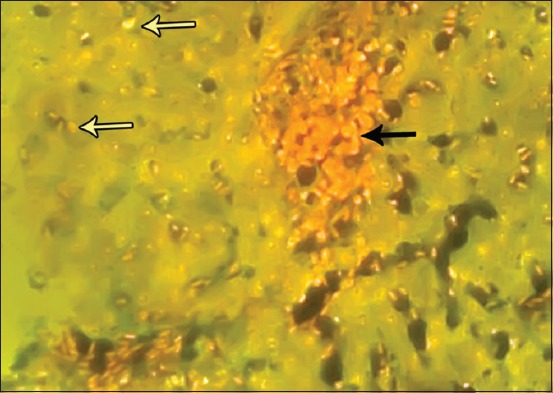
Fluorescence microscopy investigation of hepatic tissue showing the early apoptosis (yellow arrows) and late apoptotic cells as orange color (black arrow). Acridine orange and ethidium bromide stain, 100×.

The results of fluorescent microscope examination using AO/EB staining showed the occurrence of apoptosis in all hepatic tissues of birds living in polluted areas. Relatively, there was a high ratio of early apoptosis in samples collected from the area of oil fields (Figure-9), while the late stage of apoptosis or dead cells observed in the liver tissue samples was affected with a high concentration of carbon from the area of brick factories (Figure-10).

Liver is one of the most important organs to handle polluted materials in the digestive system. Furthermore, one of the most common ways of birds’ exposure to contaminated foods with carbon is their dwelling place and the environment. In the results of the current study, the cells of hepatic tissue showed that apoptosis happened because the birds were affected with accumulated carbon components in the liver parenchyma. Subsequently, CNTs are one of the major factors that induce hepatic apoptosis with various stages according to the degree and exposure period to pollutants of carbonic components.

## Discussion

The principal structure of the duck’s liver in the present work was in good agreement with several studies which described the general histological structures of liver in ostriches [17], coots [18], and turkeys [19]. On the other hand, the current histopathological results were completely identical to the findings observed in mice, in which the liver with accumulated CNTs exclusively located in the hepatic sinusoids. In addition, the CNTs rapidly cleared the blood and a fraction accreted in hepatic tissue or was eliminated by the bile [20]. Furthermore, rats treated with an amount of carbon tetrachloride can lead to hepatotoxicity. Single doses of approximately 3 ml/kg body weight showed defect in hepatic tissue ranging from congested central vein, infiltration by inflammatory cells, massive tissue necrosis, and fatty degeneration [21]. Our findings reaffirm that the liver of birds is an influential target to toxicity of carbon components, which is in line with reports by Doneley [22]. There are many compounds in pesticides (phosphorous, Vitamin D3 analogs, and metaldehyde), heavy metals (zinc, iron, copper, and lead), and some specific drugs known to be hepatotoxic in birds and other animals. On the other hand, several studies have demonstrated the effects of CNTs which have greater absorption in the gastrointestinal tract [23]. The results of these studies showed a significant modification in the fine structure of small intestine in the mice which drank water containing CNTs. They found the proliferation of epithelial cells and an increasing number of formless villi in the mucosal layer of the small intestine, most pronounced in experimental animals exposed for up to 2 months. The refined MWCNTs can induce hepatotoxicity condition in male Swiss Webster mice through oxidative stress activation [24]. Moreover, Ji *et al*. [25] reported that gene expressions for G protein-coupled receptors, tumor necrosis factor-alpha, nuclear factor-kappa B, cholesterol biosynthesis, natural killer cell-mediated cytotoxicity, and the metabolism by cytochrome P450 signaling pathway changed in the liver of mice exposed to MWCNTs. Liver is the largest organ in the body post-integument and since it is intimately associated with the digestive tract, it plays a fundamental role in the intermediate metabolism of ingested materials such as carbohydrates, proteins, and lipids. Accordingly, liver is the main powerful target organ for toxin compounds and other ingredients. In this study, we noticed that the mild and acute pathological hepatic lesion observed in the birds of polluted areas is the result of toxicity effects of different dosages of carbon nanoparticles from food consumption. The cause of all undesirable toxicity is the remnants of oil fields and brick factories, which increase carbon monoxide concentration in the atmosphere. Lamberti *et al*. [26] indicated that exposure route, shape, surface chemistry, purity, and dose of CNTs play important roles in these differential toxicities in various organs of patients with exposure to CNTs. The results of some previous authentic studies described that oxidative MWCNTs would be retained for long time in spleen, lung, liver, and other tissues after entering the body and can cause damage in these tissues through inducing inflammation, granuloma, apoptosis, and DNA damage [27]. In mice, the results indicated that exposure to oxidative MWCNTs could not induce the liver tissue to produce metallothionein that plays a role in the protection against oxidative stress and metal toxicity [28]. The results of apoptotic cells in this study are similar to those of the researchers who mentioned that CNTs induce apoptosis in the lung of mice [29] and birds [30]. On the other hand, the previous studies demonstrated that apoptosis occurs through defense mechanism like in immune reactions or when cells are damaged by detrimental agents or through disease as well as physiologically due to self-destruction of cells [31]. Apoptosis is often related to bacterial infectious diseases in animals, causing considerable morbidity and mortality. Principally, these bacterial infections are playing an essential role in triggering apoptosis [14].

## Conclusion

The carbon particles (soot) have adverse effects on the liver of birds living in polluted environments. These particles of carbon that aggregates as UFCB and MWCNTs lead to the incidence of some histopathological changes in the hepatic tissue of birds. In this study, we observed that liver morbidity of birds living in farms affected by the pollutants of brick factories is higher than that of the birds living in farms affected by pollutants of the oil fields. Prominently, the large number of death cells seen in liver samples involved in early and late stages of apoptosis is associated with carbonic components in a polluted atmosphere.

## Authors’ Contributions

AMA and AFB planned and directed the project; JGA and EAKZ performed the practical parameters in the histopathological laboratory; AMA and JGA developed the perspective framework. AFB and EAKZ composed the early draft of the article. All authors participated in the stage of discussing the results and commented on final manuscript. All authors have read and approved the final manuscript.
